# Computational modelling for decision-making: where, why, what, who and how

**DOI:** 10.1098/rsos.172096

**Published:** 2018-06-20

**Authors:** Muffy Calder, Claire Craig, Dave Culley, Richard de Cani, Christl A. Donnelly, Rowan Douglas, Bruce Edmonds, Jonathon Gascoigne, Nigel Gilbert, Caroline Hargrove, Derwen Hinds, David C. Lane, Dervilla Mitchell, Giles Pavey, David Robertson, Bridget Rosewell, Spencer Sherwin, Mark Walport, Alan Wilson

**Affiliations:** 1 School of Computing Science, University of Glasgow, Glasgow, UK; 2 The Royal Society, London, UK; 3 Improbable, London, UK; 4 Arup, London, UK; 5 MRC Centre for Global Infectious Disease Analysis, Department of Infectious Disease Epidemiology, Imperial College London, London, UK; 6 Willis Towers Watson, London, UK; 7 Centre for Policy Modelling, Manchester Metropolitan University, Manchester, UK; 8 Centre for Research in Social Simulation, University of Surrey, Guildford, UK; 9 McLaren Applied Technologies, Woking, UK; 10 National Cyber Security Centre, UK; 11 Henley Business School, University of Reading, Reading, UK; 12 Consultant, UK; 13 School of Informatics, University of Edinburgh, Edinburgh, UK; 14 Volterra Partners, London, UK; 15 Department of Aeronautics, Imperial College London, London, UK; 16 UK Research and Innovation, London, UK; 17 The Alan Turing Institute, London, UK

**Keywords:** modelling, decision-making, data, uncertainty, complexity, communication

## Abstract

In order to deal with an increasingly complex world, we need ever more sophisticated computational models that can help us make decisions wisely and understand the potential consequences of choices. But creating a model requires far more than just raw data and technical skills: it requires a close collaboration between model commissioners, developers, users and reviewers. Good modelling requires its users and commissioners to understand more about the whole process, including the different kinds of purpose a model can have and the different technical bases. This paper offers a guide to the process of commissioning, developing and deploying models across a wide range of domains from public policy to science and engineering. It provides two checklists to help potential modellers, commissioners and users ensure they have considered the most significant factors that will determine success. We conclude there is a need to reinforce modelling as a discipline, so that misconstruction is less likely; to increase understanding of modelling in all domains, so that the misuse of models is reduced; and to bring commissioners closer to modelling, so that the results are more useful.

## 1. Introduction

Computational models can help us translate observations into an anticipation of future events, act as a testbed for ideas, extract value from data and ask questions about behaviours. The answers are then used to understand, design, manage and predict the workings of complex systems and processes, from public policy to autonomous systems. Models have spread far beyond the domains of engineering and science and are used widely in diverse areas from finance and economics, to business management, public policy and urban planning. Increasing computing power and greater availability of data have enabled the development of new kinds of computational model that represent more of the details of the target systems. These allow us to do virtual *what if?* experiments—even changing the rules of how this detail operates—before we try things out for real.

Analysis and explanation are just the starting point for the utility of models. They can help us to visualize, predict, optimize, regulate and control complex systems. In the built and engineered world, manufactured products can be simulated as part of the design process before they are physically created, saving time, money and resources. Buildings, their infrastructure and their inhabitants can be modelled, and those models can be used not only to maximize the efficiency and effectiveness of the design and build processes, but also to analyse and manage buildings and their associated infrastructure throughout their whole working lifespan. In the public sector, policies can be explored before they are implemented, exposing potential unanticipated consequences and suggesting ways to prevent their occurrence.

It takes time and effort to develop good models, but once achieved they can repay this investment many times over. Just as physical tools and machines extend our physical abilities, models extend our mental abilities, enabling us to understand and control systems beyond our direct intellectual reach. This is why they will have such a radical impact: not just improving efficiency and planning, but extending to completely new areas of our lives. Computational models will change the ways we can interact with our world, perhaps allowing completely new ways of living and working to emerge.

Computational modelling is like any other technology: it is neither intrinsically good nor bad. Models can inform or mislead. Modelling can be applied well or misapplied. It is for this reason that a better understanding of the processes of computational modelling and a greater awareness of how and when models can be reliably used are important. This cannot be just left to the modellers but some of the understanding is also needed by commissioners and users of these models. Making the right decisions when commissioning a model or when and how to use a model is as important as the more technical aspects of model development. A hammer may be perfectly designed by its engineers and fit its specification exactly, but be worse than useless for driving in screws.

The contribution of this paper is to bring together current thinking about, and experiences with, computational modelling. It does not reveal new research or results, but rather aims to serve as a guide for all those involved in modelling. It is of direct interest to a range of potential stakeholders for modelling: commissioners, owners, developers and users, but it is also important for those who may be affected by the insights that come from these models in the public, private, academic and not-for-profit sectors.

Computational models are reaching into domains beyond those where they have been traditionally applied (the physical and life sciences and engineering); they are being used for new purposes; and their complexity means that they have different properties from simpler models (such as those which can be completely checked using analytic methods). This extension has the potential for new application and utility across many aspects of our collective life, but it also means there is a greater potential for their misuse: misleading as to the current state of what is modelled and informing decisions where they are not suited. Hopefully this paper will help educate all relevant stakeholders as to these opportunities and dangers, and thus help make these tools a positive force in the new areas in which they are being applied.

This paper distinguishes some of the different purposes for a model: this has a significant impact on how the model should be developed, checked and used. It gives an overview of some of the different technical bases, to provide some understanding of their nature and properties. It also looks at some of the future directions in which modelling is developing. It includes two checklists, aimed at the full range of stakeholders: to help people ask the right questions of models and modellers and hence improve the whole modelling process.

This paper is a condensation of the recent Blackett review *Computational Modelling: Technological Futures* [1] that was initiated by Government Office for Science and the Prime Minister's Council for Science and Technology. It is organized into five sections covering: where models are used, why model, making and using models, types of model and analysis and future directions. Appendix A contains two checklists: *making and using models* and *what users should ask about a model*.

## 2. Where models are used

This paper aims to bring together knowledge about computational modelling across a wide range of domains, from public and economic policy to physical systems. A few examples and observations illustrate the current breadth and scope of modelling.

In public policy, models can enhance the quality of decision-making and policy design. They can offer cost–benefit analyses of various policy and delivery options, help manage risk and uncertainty or predict how economic and social factors might change in the future. There is still considerable untapped potential in this area but also obvious dangers.

The science of urban modelling is rapidly developing, and modelling is routinely used in the retail and transport sectors. However, substantial research challenges and opportunities remain, particularly in dynamics and in deploying new data sources. Greater research coordination, and policies that make high-quality urban models available to local authorities, could help to realize the tremendous potential of ‘urban analytics’.

Models play crucial roles in finance and economics, from identifying and managing risk to forecasting how economies will evolve. Yet major changes are afoot in economic modelling, triggered by the global economic crisis, the availability of huge datasets, and new abilities to model people's behaviour that overturn old certainties.

In business and manufacturing, models underpin a wide variety of activities, enabling innovative high-quality design and manufacturing, more efficient supply chains and greater productivity. Modelling can also improve businesses' organizational efficiency, commercial productivity and profitability. In manufacturing, models tend to fall into three broad categories: complex models aimed at modelling physical reality with a high degree of accuracy, reduced physical models that capture behaviour at a specific scale and representative models (so-called ‘black box’) models that fit data and trends.

Finally, environmental modelling, including climate change, plays an important role in guiding government policy as well as business decisions, in situations ranging from noise reduction to flood risk assessment and wherever there is an opportunity to enhance social resilience to severe natural hazards. Open-access datasets are particularly useful in this domain.

## 3. Why model

Given the effort it takes to make and check a good model, how might one decide whether this effort is worthwhile? For a given system, there are a number of answers to this question:

— The complexity of the system means that the risks and consequences of any choice cannot be anticipated on the basis of common sense or experience.
— There may be too many detailed interactions to keep track of, or the outcomes may be too complicated and interwoven to calculate easily.
— It is infeasible or unethical to do experiments with the system.
— One needs to integrate reliable knowledge from different sources into a more complex whole to understand the interactions between them.
— There is a variety of views from stakeholders or experts about a complex system they are part of, which needs bridging in order to come to a coherent decision or find a compromise.
— One needs to be prepared for possible future outcomes in a complex situation.

The variety of answers are indicative of the different purposes a model may have.

### 3.1. Purposes

The purpose of a model affects how it should be developed and checked and, crucially, it informs potential users as to how they should judge a model and from that what it can be reliably used for. Thus identifying the different uses for a model is very important. Here, we distinguish five broad categories of model purpose—there are many others (e.g. those listed in [2]), but the following five cover many of the main scientific purposes (the first two empirical, the last three theoretical).

#### 3.1.1. Prediction or forecasting.

Almost all computational models ‘predict’ in the weak sense of being able to calculate an anticipated result from a given set of variables. A stronger form of prediction goes further than this, anticipating unknown (usually future) outcomes in the observed world (some describe this as ‘forecasting’). This sort of prediction is notoriously difficult for complex systems, such as biological or social systems, and thus claiming to be able to forecast for these systems may be misleading. If we truly do not know what is going to happen, it is better to be honest about that, rather than be under a false impression that we have a workable prediction. Fitting *known* data (e.g. ‘out of sample data’) is not prediction in this sense.

#### 3.1.2. Explanation or exploration of future scenarios.

Particularly when considering very complex phenomena, one needs to understand why something occurs—in other words, we need to explain it. In this context, explanation means establishing a possible causal chain, from a set-up to its consequences, in terms of the mechanisms in a model. This degree of understanding is important for managing complex systems as well as understanding when predictive models might work. With many phenomena, explanation is generally much easier than prediction—models that explain why things happen can be very useful, even if they cannot predict reliably the outcomes of particular choices. For example, a social network model may help explain the survival of diverse political attitudes but not predict this [3].

#### 3.1.3. Understanding theory or designs.

This usually involves extensive testing and analysis to check behaviours and assumptions in a theory or design, especially which outcomes are produced under what conditions. Outcomes can be used to help formulate a hypothesis; but they can also be used to refute a hypothesis, by exhibiting concrete counter-examples. It is important to note that although a model has to have some meaning for it to be a model, this does not necessarily imply the outcomes tell us anything about real systems. For example, many (but not all) economic models are theoretical. They might include assumptions that people behave in a perfectly rational way, for example, or that everybody has perfect access to all information. Such models might be later developed into explanatory or predictive models but currently be only about theory.

#### 3.1.4. Illustration or visualization.

Sometimes one wants an illustration or visualization to communicate ideas and a model is a good way of doing this. Such a model usually relates to a specific idea or situation, and clarity of the illustration is of over-riding importance—to help people see (possibly complex) interactions at work. Crucially, an illustration cannot be relied upon for predicting or explaining. If an idea or situation is already represented as a model (designed for another purpose) then the illustrative model might well be a simplified version of this. For example, the DICE model (dynamic integrated model of climate and the economy) is a ‘simplified analytical and empirical model that represents the economics, policy, and scientific aspects of climate change’ [4]. This is a simpler version of the RICE model [5] that is used to teach about the links between the economy and climate change.

#### 3.1.5. Analogy.

Playing with models in a creative but informal manner can provide new insights. Here, the model is used as an aid to thinking, and can be very powerful in this regard. However, the danger is that people confuse a useful way of thinking about things with something that is true.

If the purpose of a model is unclear or confused, this can lead to misunderstandings or errors. To give two examples, a theoretical model might be assumed to be a good way of thinking about a system, even though this might be crucially misleading, or a model that helps establish a good explanation be relied upon as a predictive model. Making clear the purpose of a model is good practice and helps others know how to judge it and what it might be reliable for.

## 4. Making and using models

Models have many technical aspects, such as data, mathematical expressions and equations, and algorithms, yet these are not sufficient for a model to be useful. To get the best out of a model, model users and commissioners must work closely with model developers throughout its creation and subsequent application.

### 4.1. Asking the right question

It is important to make sure that a model is dealing with the right issue and helping to ask the right question. Even a high-quality model will not be helpful if it relates to an issue that is not the main concern of the user. Conversely, asking a model to answer more and more detailed questions can be counterproductive, because it would require ever more features of the real system to be included in the model. In other words, models need to be ‘requisite’—they must have an identified context and purpose, with a well-understood knowledge base, users and audience, and possibly developed within a particular time constraint [6].

### 4.2. Who does what?

Although a very simple model might be the work of one person, usually a team of people will be involved, and it is important to be clear about the individuals' roles. There will be at least an owner, or *commissioner*: the person whose responsibility it is to specify what the model is expected to do, provide the resources needed to get the model built, and sometimes monitor how the model is used. There will be *model developers*, whose job is to design, build and validate the model; and analysts who will generate results from the model. Developers and analysts are often, but not always, the same people. There will also be the *model's users*: those who have the problem or question that the model is designed to answer. And it is good practice to have a *reviewer* or quality assurer, someone independent from the team whose task is to audit the model and the way it has been developed to ensure that it meets appropriate quality standards and is fit for purpose—standards will vary according to the importance and risk of the area. Each of these roles may be carried out by several people—a large model might need a team of developers, and the review might be carried out by a group of peer reviewers, for example. In all but the most modest models, however, there should be at least one person for each role, because the skills required for each are different.

### 4.3. Specifying a model

Sometimes it is possible to be precise about what a model should contain, before the model is created. One can then write a specification and hand it over to a group of professional model developers. This situation can arise when dealing with a logistical or operational question, where there is a great deal of certainty about the system and clarity about what the model should output. Much more often, however, the situation to be modelled is complex; the processes to be modelled are uncertain; and the questions to be answered are vague. In such cases, model commissioners need to stay very close to the modelling process, getting involved in an iterative process of deciding what should be included and how it is represented. Such models will often produce a range of results and may identify possible tipping points. This is usually the best approach if one is concerned with strategic or policymaking questions; dealing with one-off issues; addressing uncertainty about the consequences of actions; or is unclear about appropriate ways of judging what a system does. In these cases, those involved in the process need to exercise their collective judgement when interpreting the results.

### 4.4. Finding the data and assessing quality

All too frequently, one does not discover exactly what data one needs until the model has been built, so it often becomes an iterative process of finding data and developing the model. However, there are a few helpful distinctions to be made that will enable a model commissioner to ask model developers the right questions. The first distinction is between the data needed to specify and build the model; the data that will be used to check the model's output; and the data needed for day-to-day use of the model. The second distinction concerns the levels at which the model operates: the micro-level, describing how the smallest components of the model behave (for example, the cars in a traffic model); the meso-level, describing how the components are linked together (for example, the road layouts); and the macro-level, covering the properties of the system as a whole (for example, the funding for new road infrastructure). The micro-level may be determined by the science behind the model, by qualitative evidence, or by ‘big data’ analyses. The meso-level might reflect the structure of the system. And the macro-level may include data such as aggregate statistics over a long period of time. Sometimes it is acceptable to use closely related proxies for these data.

For models that are intended to explain or predict the outcomes of processes that take place over time, we usually need data that have been collected over a period (referred to as time-series data, or longitudinal data). However, such data are often difficult to obtain, not least because of the time it takes to gather the dataset, but also because definitions may have changed in the intervening period, making data points measured at different times not strictly comparable. Also, if one is using data collected at two points in time from the same individual or organization, one must consider the effects of those who stop participating during the data collection period, which may lead to a biased sample.

### 4.5. Building a model

Designing and building a model has some of the characteristics of software development and many of the same techniques and tools can be used. There are two basic approaches: either one can attempt to specify in detail what the model should do and then construct it to match that specification; or one can build the model in a much more iterative fashion, starting with a basic model at an early stage and incrementally improving it, meanwhile checking that it matches the users' requirements. These requirements may themselves change as the users improve their understanding of the problem.

Model building is often out-sourced to consultancies or is the responsibility of specialized teams of in-house developers. The downside of out-sourcing is that barriers to communication may arise, especially when the commissioner and the developer are in different organizations with different cultures and different priorities. Regardless of the development approach and the location of the developers, it is essential that design decisions are logged and the development process is documented (not just the final modelling outcomes). This documentation will be an important input into the model's quality assurance review. It is important to establish, at the start, to whom the resulting model code belongs.

### 4.6. Documenting a model

A model will be all but useless if it lacks appropriate documentation. Several different kinds of documentation are needed:

— Documentation of the model code, sufficient to explain in detail what it does and how it does it. Some of this will be integrated into the code as comments, but there will also need to be separate documents intended for developers.
— Documentation aimed at analysts, who may want to change model parameters but not the model code. Such documentation will need to explain how to run the model, the computing system it needs, supporting software if any, and the various files that the model requires as inputs and generates as outputs.
— Documentation for users. This may include presentations, tutorials and user guides aimed at people who want to use the model but do not need to know about its mechanics. While the documentation should be comprehensible to non-experts, it should include an explanation of the assumptions on which the model is based, as well as its objectives and limitations.

Documentation takes time to prepare, often more time than building the model itself. But it is essential, because the original developers, reviewers, users and even the commissioner may move on to other roles, taking their knowledge and expertise with them. Moreover, if a decision that relies on the model is challenged, internally or externally, by public opinion or judicial review, the documentation may have legal significance.

### 4.7. Quality assurance

*Validation* asks the question: have we built the right model, i.e. is the model a suitable representation of what is being modelled? This often involves testing the model against known data or behaviours, to demonstrate that the model is faithful and gives the expected outcomes. *Verification* asks the question: have we built the model right? This means checking the model itself, for example, checking we have the correct formulae in all the spreadsheet cells, or checking how errors and uncertainties propagate and for which inputs the results are undefined.

### 4.8. Uncertainty

There are many ways in which uncertainty can arise. These include: errors in measuring or estimating; inherent chance events in the system being modelled; an underappreciation of the diversity of events in a system; ignorance about a key process, such as how people make decisions; chaotic interactions in the system such that even a small change can switch behaviours into another mode; and the complexity of the model's behaviour itself, which model developers may not fully understand. It is important to consider the uncertainties in the data that underpin a model, and the level of uncertainty that might be acceptable in the model's answers. In addition, there may be considerable uncertainty about the basic mechanisms that are being represented in the model and about whether alternative models using quite different mechanisms might be better. Moreover, a complex model can sometimes act as an ‘uncertainty amplifier’, so that the uncertainty in the results is much greater than the uncertainty in the setup of the model and the data it uses. Just as there are different kinds of uncertainty that affect a model, there are different kinds of uncertainty in model outcomes. The answers a model gives might be basically correct, but somewhat prone to a degree of error. In other cases, the outcomes might suddenly vary sharply when the inputs change, or shift from a smoothly changing continuum to an ‘on/off’ outcome. The kinds of uncertainty in model outcomes affect how it can be used reliably. Consequently, it is vital that the uncertainty in a model's results is communicated together with the main results.

### 4.9. Communicating a model

While the process of modelling can greatly increase one's understanding of a problem, the true value of a model only becomes apparent when it is communicated. The communication of model results is an important part of the modelling process: the user interface or visualization is the only contact those not directly working on it will have with a model. A visualization should encapsulate all that is important to know about the underlying model. It must somehow communicate the model's results and (ideally) its assumptions to the intended audience, who may base important decisions on their understanding of the visualization. Consequently, even at the scoping stage it is crucial to consider who the user of a model will be, and how they will want to interact with it.

Making educated simplifications and assumptions is an inherent part of the modelling process, as is the presence of some uncertainty in model results. Given the compelling nature of well-designed visualizations and user interfaces, it is vital that they do not misrepresent the reliability of the results they communicate, just as an executive summary should be representative of the conclusions and caveats of the underlying report.

### 4.10. Maintenance

As the *Review of quality assurance of government models* (commonly known as the Macpherson review) found in 2013 [7], once a model exists, it may be used for purposes beyond that for which it was originally designed, and it may continue to be used long after the time when it should have been replaced. There are at least three reasons for this:

— Users are reluctant to abandon the model. Unless appropriate maintenance activities have not been put in place, the model's results may become less and less accurate because the system being modelled has changed. The fact that the model has been successful in the past can bolster confidence in its credibility, without anyone realizing that the model no longer fits what it is modelling.
— The model's use has changed. While the model would have been tested to give good results for its original purpose, the quality assurance may not guarantee its validity following ‘creep’ in the way it is being used. In addition, as staff involved in the model move on to other projects, the original understanding of the model's assumptions, scope and limitations may get lost.
— Model accretion. If extra parameters or routines are added to the model to deal with new demands or new data, the model may eventually become so complicated that it is difficult for anyone to understand it and use it correctly.

These dangers can be avoided, or at least ameliorated, by scheduling regular reviews of the model to check that it remains fit for purpose, and to ensure that the documentation remains relevant. The review may conclude that the model should be retired or re-written. To ensure that such reviews do take place, models should have long-term owners with responsibility for their continued maintenance.

### 4.11. Preserving a model

An important aspect of documenting and maintaining a model is to ensure that it is properly preserved for later access, regardless of institutional and personnel changes and the evolution of computing infrastructure. One increasingly popular solution is to make the model and documentation open source and lodged on a platform such as GitHub (https://github.com/) or CoMSES (https://comses.net/). ‘Open source’ means that the model code is freely available and publicly accessible, under an open licence. Open sourcing a model also means that others can modify and use the model for their own purposes (including, depending on the licence conditions, for commercial purposes). The advantages of open source include that what the model does and how it does it is freely accessible and ‘transparent’; other users and modellers can assist in the development and maintenance of the model, and that the platform takes over responsibility for the model's long-term preservation. On the other hand, opening up a model in this way can raise issues of commercial confidentiality and individual privacy and data protection. The latter can be especially tricky if the model depends on data provided by individuals for its calibration.

## 5. Types of model and analysis

One might not need to know anything about the mechanisms inside a very well established and understood model. However, for other models (especially newly developed models) it is useful to have some understanding of the basis of their construction. In this section, we give a brief summary of the main aspects and approaches used.

Stakeholders often have very different perspectives on the key abstractions and assumptions about the system being modelled. Frames of reference [8] are one way of articulating the variety of perspectives, and their context. Clarity on frames allows different levels and type of concern to be balanced within model development and analysis, driving the selection of model type and techniques. Some common frames are the following.

*Geographic*: spatial and topological relationships, such as (static) locations of adjacent underground stations and the positions of emergency exits, or (dynamic) flows in a pipeline and networks of sensors on people, animals and objects.
*Temporal*: how the expected certainty of the model varies over time. For example, weather forecasting becomes less certain the further we look into the future, and navigation models become less precise as we move away from the position where we last verified our location.
*Physical*: underlying natural science, ecosystems and their governing laws, such as those that govern water flow, heat transfer or atmospheric physics.
*Security*: threats and their mitigations, such as access controls, which prevent unauthorized persons or systems from physically entering or digitally accessing a system, and encryption methods that encode data so they can only be accessed via keys.
*Privacy*: anonymity, identity, authentication of personally identifiable information, and controls on intended and unintended disclosures.
*Legal*: obligations, permissions and responsibilities for different components within the system and for human users of the system.
*Social*: communication and interaction relationships between humans involved in the system, and between humans and the physical/natural world and the underlying technologies.
*Economic*: quantitative aspects of resource consumption, production and discovery; typical resources are energy, money and communication bandwidth.
*Uncertainty*: what the acceptable bounds of uncertainty are for various aspects of the system, and how bounds are qualified, quantified and related to each other.
*Failures*: relationships between components that can fail or operate incorrectly, including fail-safe mechanisms and redundancies.

Each frame (or frames) may require a different type of model and analysis, and all kinds of framing demand judgements about the scales to be adopted, from the coarse to the fine-grained. A model developed to address one frame of reference may not be suitable for another frame and can be positively misleading if this is attempted. For example, using a costing model for rail ticket sales to assess the order in which to upgrade signals or the impact of lengthening trains by adding carriages could give very misleading results. This is because the costing model would not include details of how signals depend on each other, or the loads that rails are designed to withstand. It is thus helpful to make these frames of reference explicit when developing or commissioning models.

### 5.1. Types of model

There are a wide range of computational modelling techniques, but they differ principally along a few dimensions. Selecting particular points along these dimensions implies a set of abstractions and assumptions about the system being modelled, which in turn determines how observations are represented.

*Non-deterministic* models can deliver several possible outputs from a given set of inputs. If you run a non-deterministic model today, and then run it again tomorrow with the same inputs, you may obtain different answers.
*Deterministic* models always produce one specific output from a particular set of inputs or initial conditions. Determinism in models is often highly valued, because it allows one to make absolute assertions. However, many aspects of the physical world and human behaviours are fundamentally non-deterministic, and it may not be useful to try to model them in a deterministic way.
*Static* models have no inherent concept of time and so outputs do not change over time. For instance, spreadsheets are static models, unless they explicitly encode time as an input.
*Dynamic* models have outputs that change over time. Ordinary [9] and partial differential equations [10] are common mathematical dynamic models for representing the rate of change over time; they are widely used in engineering and environmental monitoring, and also in finance and economics. System dynamics [11] is a technique based on ordinary differential equations that is used widely in business and government when considering new policies. It is used to explore the possible effects of different policies and unanticipated consequences, as well as develop understandings of the structural source of general patterns of behaviour.
*Discrete* models represent objects or events by values that go up in steps—a series of integers or characters, for example. Common discrete models are based on sets of discrete states; for instance, transition systems [12] consist of discrete states with transitions between them.
*Continuous* models involve representations that are ‘smooth’ and ‘dense’, using real numbers, for example. Differential equations are common continuous models. It is possible to combine both discrete and continuous aspects into a single model. For instance, a model may consist of a finite number of discrete states with the rates of transition between the states being continuous.
*Stochastic* (also called probabilistic or statistical) models [13] have an inherent element of random, or uncertain, behaviour and the events are assigned probabilities. This can be viewed as a special case of a non-deterministic model in which the probabilities are known.
*Individual-based* models represent each individual explicitly. These models are useful when one needs to track each individual through a system, or individuals vary significantly in their behaviour, or together the individuals form a complex and emergent system whose behaviour cannot be derived from simple aggregation. Typical examples include social insects, extremely large telecommunications networks (including the Internet), transportation networks, and stock markets. These systems are often tackled using *agent-based* models [14], typically containing a large set of autonomous agents that each represent individuals that interact with each other based on their individual attributes and behaviours.
*Population* models collectively represent large groups of individuals and are useful when individuals do not vary and an individual-based model is not tractable. When individuals do vary, but according to a small number of attributes, a population model based on *counter-abstraction* [15] that records the number of individuals with each trait (or combinations thereof) may be suitable.
*Logic* models are statements in a formal logic, which may range from classical predicate logic [16], to temporal logics [17] for future behaviours, and probabilistic temporal logics [18] for future certainties/uncertainties.
*Automata and process algebraic* models [19,20] allow simple and elegant representations of events occurring in multiple processes that send messages to each other. The underlying languages are algebraic, which means there are laws that define how the different operators (a sequence or choice between events, for example) relate to each other.
*Black-box* models fit data and trends without revealing internal workings. Machine learning [21] is a common technique based on algorithms that, in effect, learn directly from past examples, data and experience. Machine learning is most valuable where there is little prior knowledge or intuition about how a system works, but where there is considerable available data. This opens up the possibility of making predictions about the future by extrapolating patterns in the data, in domains where that has not previously been possible. At present, the results may be difficult to interpret or explain; and the models may be robust only within relatively narrow contexts.

Common example combinations of techniques include stochastic partial differential equations and hybrid automata [22]. The latter have discrete states and transitions between them, and each state is a set of differential equations that describes the continuous behaviour that applies during that state. A drawback of some combinations is that analysis can be difficult and may be poorly supported by automated tools.

### 5.2. Ensemble modelling

Ensemble modelling is an important approach to model combination that involves running two or more related (but different) models, and then combining their results into a single result or comparing them. When results within the ensemble disagree, this can contribute to an understanding of whether uncertainty is present as a result of the type of model (and so the choice of model is crucial), or exists within the system. As an example, ensembles are widely used in weather forecasting, to show the different ways in which a weather system can develop.

### 5.3. Analysis

Just as there are many types and techniques, there are also different ways to ask questions and obtain answers from models. Often the questions one can ask are fundamentally linked to the modelling technique. One of the most common types of analysis is simulation, usually over a time period, often called ‘running’ the model. If the model is deterministic, there is only one simulation result; the output of a static model depends entirely on the values assumed for any input parameters. But if the model is non-deterministic (i.e. has a random element) then there are many possible answers—each time you run it you will get a different answer that reflect random elements in the choices or in the environment. If you have such a model it will require many runs to achieve a representative picture of what happens.

Another type of analysis uses logic to formulate questions and reasoning techniques to answer them. For instance, questions about the performance of a modelled telecommunications service such as *after a request for a service, is there at least a 98% probability that the service will be delivered within 2 s?* can be expressed in a probabilistic temporal logic. Automated reasoning tools such as theorem provers and model checkers can be used to derive the answer.

### 5.4. Role of data

Data are observations that can provide evidence for a model. The exact role of data depends on how they were obtained, and the purpose of the model. For example, if the model aims to offer rigorous explanations or predict future outcomes of an existing system, then data are necessary to validate the model. If, on the other hand, the purpose of the model is to specify a system design, or define how an intended system is required to behave, then data are used to validate the system against the model. In other words, after the system has been implemented, one checks it behaves as it should.

There is a further role for data when we are confident about the essential structure of the model, but do not know the bounds of some parameters. In this case, data are used to fine-tune parameters such as the duration or speed of an event. In all cases, care and expert judgement about interpreting validation results is required, especially when the model has been determined mainly by data with few structural assumptions imposed by the modeller, or if the data are sparse, or when it is not possible to experiment with the deployed system. For example, air traffic systems are so crucial to modern life that one cannot experiment with various parameters—such as frequency of landings or proximity of aircraft—to comprehensively check the system against the model.

## 6. Future of modelling

Modelling is changing fast, due to rapid growth in computing power, an explosion in available data, and greater ability of models to tackle extremely complex systems. In the future, there will be a greater need for reliable, predictive models that are relevant to the large-scale, complex systems we want to understand or wish to construct. While larger and more sophisticated models will add to predictive capability, they will also allow us to get a better grasp on the limits to prediction, fundamental uncertainties, and the capacity for tipping points and lock in. Some models will work closely with (perhaps be embedded in) operational systems and derive data from them, potentially in real time. These data may come from the many sensors and actuators that are now being added to systems, and we will see new forms of modelling emerge as a consequence. The following offers a glimpse of the changes, challenges and potential rewards over the coming decade.

— *Large-scale availability of data about individuals will transform modelling.* When we model a population of individuals today, we often attempt to make predictions using aggregate models based on assumptions about hypothetical, ‘average’ members of the population. In future, it may be easier to eliminate these assumptions by modelling the individuals directly.
— *Models will require more extensively linked data.* Some data may be derived not from measurement but from other models, requiring additional links to derived data.
— *Modelling will span many scales, and many levels of detail.* As various modelling communities come together, bringing expertise from different disciplines and sharing approaches to model design, we will see more sophisticated ways to link models in ways that describe systems at multiple levels of detail.
— *More models will be built by computers.* Models may be constructed from data by automated or semi-automated inference. These models will have the capacity to reveal unexpected results, but it may be hard to guarantee that their mechanisms continue to operate reliably in the face of new evidence.
— *Models will help to train computers.* When computers learn from real-world data, they need to be exposed to both positive and negative examples. The latter can be difficult to find: models may be able to generate verisimilar data representing failures.
— *More systems will become part of models and more models will become part of systems.* More components of engineered systems will be software: that software may be incorporated into models used to predict the behaviour of the aggregate system built from components and embedded models may drive aspects of system behaviour. This will change the dynamic between modelling and deployment of systems.
— *New technologies will change modelling paradigms.* Specialist quantum simulators will soon become available. They may allow us to develop models that predict properties of materials or pharmaceuticals, or make scenario planning for finance, defence and medical diagnosis more tractable.
— *Ubiquitous sensors will require new forms of modelling.* Sensors, actuators and processors are becoming more ubiquitous and more intelligent, yet sensors decalibrate and degrade over time both individually and as networks. The unreliability of data from sensors will require more spatial, dynamic and probabilistic styles of modelling.
— *Modelling will be used more often for strategic and policy-level issues.* Modelling will increasingly be used for high-level organizational planning and systems thinking, adding more detail to potential future scenarios, and allowing analysis of possible outcomes of policy interventions.
— *Senior decision-makers will increasingly become involved in modelling.* Senior decision-makers will participate more often in building and using models. A willingness to engage directly in modelling, for example, by bringing modelling into the boardroom, will increasingly be seen as a sound approach to managing complexity.
— *Some models will be oriented more towards humans and their personal characteristics.* We will have a greater opportunity, as individuals, to supply (personal) data that could be used to stimulate modelling. However, there remain deep, unresolved social and ethical issues around the ownership of data and the use of models derived from personal data.
— *Models will help to train humans.* Simulators are already used to train jet pilots, Formula One drivers and veterinary surgeons. High-fidelity models will soon be used more widely, in conjunction with virtual reality and ‘gamification’ in training for doctors, military personnel, police forces and school pupils, to name just a few.
— *Models will become an important way to understand properties of many complex systems.* We increasingly build systems so complex that their behaviours cannot be explored in any depth. The Internet itself is an example of a complex, engineered system on which much of our developed world now depends, and which is continuously modelled and monitored in order to explore its behaviours and monitor its performance.

## 7. Summary and conclusion

In order to deal with an increasingly complex world, we will need ever more sophisticated models. Computational models have the potential to help us make decisions more wisely and to understand the complicated and often counter-intuitive potential consequences of our choices. However, as with all tools, they can be applied in wrong or misleading ways. Thus a degree of understanding of their uses and properties is desirable. This paper brings together some of that knowledge in order to promote the better understanding of models. This is summarized by four points.

*First*, it is important to be aware that models have different kinds of uses. Effective deployment requires both the user and the modeller to be aware of their capabilities and limits. We have outlined some broad categories of model purpose and the key role that framing plays in balancing perspectives and getting the best out of a model. Confusing or conflating model purpose can result in the inappropriate use of models, or a mistaken assessment of their reliability.

*Second*, creating and using models well involves far more than raw data and technical skills. A close collaboration between model commissioners, developers, users and reviewers provides an essential framework for developing and using an effective model. We have offered a guide to that process, which is vital for building confidence in any model; the checklists in appendix A suggest some questions to aid those developing models and to aid communication between the different actors.

*Third*, a little knowledge of the different technical basis on which models are built can be helpful. The multitude of different modelling techniques can often appear overwhelming; we have offered a simple introduction to some of these, explaining the various questions they can answer, and outlining their strengths and weaknesses.

*Last*, modelling is changing fast. This presents a range of future opportunities, which could transform policymaking and business operations. We have outlined some of those opportunities and also the fresh challenges they provoke. There is a consequent increasing need for the new skills and collaborations that will underpin the future of modelling.

As the power and use of modelling grows, there is increased risk that models could be poorly constructed, misused or misunderstood. We need to reinforce modelling as a discipline, so that misconstruction and misuse are less likely; we need to increase understanding of modelling across a wide range of domains, from social policy to life sciences and engineering, as well as encourage sharing of insights and best-practice across these domains; and we need to bring commissioners closer to modelling, so that results are more useful. As computational modelling develops and extends to new application areas, there is enormous potential for interdisciplinary and intersectoral developments. The cross-fertilization of ideas between industries, and academia, along with a mutual appreciation of different sectors' needs in modelling skills, represents an exciting future for computational modelling. Computational modelling already has an increasing impact on how science is done, but this will now extend into other areas of our lives. Thus it is imperative that this tool is used appropriately and carefully. We hope this paper will prompt all those involved to think about how models are used and when they can be relied upon.

## Data accessibility

This article has no data.

## Appendix A

**A.1. Making and using models: a checklist**

This checklist is inspired by the UK government's *Scope development checklist* [23], and includes some of the questions that need to be answered before and during the creation and use of a model. They could form the basis for an initial discussion between model commissioners and model developers, to clarify their understanding of what will be involved, and during model building and use. In addition, they can serve as a point of departure for model reviewers.

*Purpose*

— What is the issue or issues under consideration?
— If there is more than one issue, how are they related?
— What is the context of the issue?
— What are the specific questions that need to be answered and can modelling address them?

*Scope*

— What must the model cover?
— What can be excluded from the model?
— What is the minimum viable scope that can be used as a starting point for the model?

*Output and follow up*

— What kind of outputs or results might answer the questions raised?
— What format should be used to present the results?
— What controls are in place to make sure the model is not used incorrectly?

*Design and building*

— What level of detail is needed for the model in each of its frames of reference?
— What accuracy is required in the output?
— What should the trade-off between accuracy, simplicity and robustness be?
— What modelling techniques will be used, and why those? Which alternatives were considered?
— How do the chosen modelling techniques have an impact on the accountability of decisions?

*Data and assumptions*

— What data are available and how robust are they?
— Are there judgements about the quality of the data that will need to be made?
— How accurate are the available data, and how does that match with the required accuracy of the outputs?
— How will each of the assumptions be justified?
— What alternative assumptions could be made?

*Quality assurance*

— What verification procedures will be used to check that the model works as expected?
— How will the model be validated, and what data will be used for doing so?
— Is there a schedule of reviews to ensure that the model remains up to date?

*Who*

— Who will be the users of the model?
— Who will have overall responsibility for the model, its development and its use?
— Who will provide the data and the knowledge required to build the model?
— Who will develop the model?
— Who are the stakeholders (in other words, who is interested in the issue, who could contribute, who can influence and who will be impacted)?
— How will stakeholders be involved, and at what stage they can be most useful?
— Do the stakeholders all have the same concerns and questions about the issue? If not, what are their perspectives, and which frames of reference are to be considered?
— Who will provide quality assurance?
— Who will determine when the model is no longer useful?

*Communication*

— What methods will be used to communicate with users?
— What are their needs and abilities to appreciate the model and what it provides?
— Are visualizations, dynamic graphs and movies appropriate to convey the messages of the model and, if so, have resources been set aside to create these?

*Resources*

— Has anything similar been done before? If so, what can be learned from it?
— Is there a schedule of reviews to ensure that the model remains up to date?
— Are sufficient skills and expertise available and, if not, how can this be managed?
— What is the timescale for the work?
— What resources (time and money, for example) are available?
— Is it necessary and affordable to build a model, or could some other approach be used that requires fewer resources?
— What would be the consequences if the work is not carried out at all, or the start is delayed?

**A.2. What users should ask about a model: a checklist**

These questions are ones that those that are contemplating the use of an existing model should ask themselves. The checklist is based on the authors' experience and sources such as [23].

— Does the model offer answers to the problems that I have?
— Are the assumptions it makes ones that I agree with?
— If the model offers an explanation or prediction, has the model been validated sufficiently against empirical data (or in any way at all)?
— If the model has no or weak empirical basis, is this adequate to my needs?
— Is the model documented so that I can understand how it works?
— Is the model output clear and comprehensible?
— Does the model output seem plausible when compared with other sources of information?
— Has the degree of uncertainty in the model output been properly recorded and its implications recognized?
— Is the model being used for its original intended purpose or, if not, is the new purpose compatible with the design of the model?
— Have other stakeholders or users been involved in the model design and use and, if so, do they agree that the model is useful?
